# Exploring the impact of myotonia on daily functioning in myotonic dystrophy: a patient-reported survey

**DOI:** 10.1186/s12883-026-05002-4

**Published:** 2026-06-16

**Authors:** Valeria A. Sansone, Utkarsh J. Dang, Linda Edmondson, Jordi Díaz-Manera, Nikoletta Nikolenko, Emma-Jayne Ashley, Guillaume Bassez, Cynthia Gagnon, Federica Montagnese, Homira Osman, Céline Tard, Ulrike Nowak, Christopher Turner, Alla Zozulya-Weidenfeller

**Affiliations:** 1 The NeMO Clinical Centre, Neurorehabilitation Unit, University of Milan, Milan, Italy; 2 Carleton University, Ottawa, Canada; 3 Linda Edmondson Medical Communications, Bognor Regis, UK; 4 John Walton Muscular Dystrophy Research Centre, Newcastle University, Newcastle Upon Tyne, UK; 5 Institute of Neurology, University College London, London, UK; 6 Cure Myotonic Dystrophy UK Charity, Whitton, UK; 7 APHP, Institute of Myology, Paris, France; 8 Clinique des Maladies Neuromusculaires, CIUSSS Saguenay–Lac-Saint-Jean, Longueuil, Canada; 9 Neurologie München Nord, Munich, Germany; 10 Muscular Dystrophy Canada, Toronto, Canada; 11 Centre Hospitalier Régional Universitaire de Lille, Lille, France; 12 Admedicum, Cologne, Germany; 13 University College London Hospitals NHS Foundation Trust, London, UK; 14 Lupin Neurosciences, Zug, Switzerland

**Keywords:** Myotonia, Myotonic dystrophy, Disease burden, Symptom assessment, Patient questionnaire survey

## Abstract

**Background:**

Prominent symptoms in myotonic dystrophy (DM) negatively affect the hands/arms (DM1), legs (DM2), or relate to fatigue. Myotonia is experienced by 90% of people with DM1 and is generally the DM hallmark. However, it is unclear how people with DM consistently differentiate the impact of myotonia from other symptoms – especially weakness – across the broad framework of DM.

**Methods:**

ENSA (revEal the burdeN on daily life for myotonic dyStrophy patients due to myotoniA) was a global (targeted at eight major countries), anonymised, online, patient-reported survey to investigate perceptions of daily impact/burden of myotonia and explore antimyotonic treatment access. Thirty-two questions covering DM symptoms, broad disease impact, specific impact of myotonia and treatment formed an online survey. Internal consistency of multi-item myotonia-related domains was assessed.

**Results:**

*N=*386 (18–82 years; 62% women; 74% self-reporting a genetically confirmed DM1 diagnosis) completed ENSA. Common symptoms were hand/arm myotonia and upper-body muscle weakness (DM1, mean scores, 3.5; possible range, 1–5) and lower-body muscle weakness (DM2, mean score, 4.2; possible range, 1–5). Leg-myotonia scores increased in higher-age brackets: DM1/DM2, mean 2.86/3.71 in <40 years; >3.26/>3.97 in ≥40 years. Some respondents associated “myotonia” with DM symptoms not clinically linked to myotonia. Nevertheless, heightened “myotonia” scoring was associated with heightened daily impact/burden, and worsened abilities to undertake normal activities. Cronbach’s α was 0.96 (95% CI: 0.95–0.97) and 0.93 (95% CI: 0.91–0.95) for DM1 and DM2, respectively, indicating good internal consistency for questions assessing impact of myotonia on daily activities. 193/247(78%) DM1 and 57/83(69%) DM2 respondents were not on an antimyotonic. Treatment levels were highest in Italy (37%), where greatest treatment-related improvements and lowest dissatisfaction scores were reported (mean 24.6%; mean 40.0% for the other target countries).

**Conclusions:**

ENSA data indicate that neuromotor function is impaired and that myotonia is frequently burdensome in DM. Some patients have unmet treatment needs, although antimyotonic treatment may reduce the impact of myotonia and, consequently, improve aspects of daily living. Patients may need guidance to differentiate “myotonia” from other muscle symptoms. Nevertheless, ENSA enhances understanding of patient-reported symptom burden in DM and indicates support for antimyotonic treatment.

**Supplementary Information:**

The online version contains supplementary material available at 10.1186/s12883-026-05002-4.

## Introduction

Myotonic dystrophy (DM) types 1 and 2 are rare multisystemic disorders [1–5]. DM1 may emerge from the prenatal period through to late adulthood [5] and typically presents with muscle wasting, weakness and myotonia [1–7]. In DM1, myotonia mostly occurs in the jaw, tongue, trunk and/or distal-limb muscles [3–5], whereas DM2 commonly manifests with proximal myotonia or limb-girdle dystrophy between the third and fourth decades [8, 9]; in DM2, muscle pain worsens and myotonia develops in distal-limb muscles over time [3]. Apathy, poor disease awareness and multiple symptom manifestations mean that DM and its symptoms are largely under-reported [4, 5].

Studies in DM1 (PRISM-1) and DM2 (PRISM-2), undertaken to determine the most critical life-affecting symptoms, indicated that myotonia was a prevalent symptom, affecting 80–90% of patients [10, 11]. Whereas the heterogeneity of the DM phenotype is well characterised using patient-reported tools [12–14], little is known about the specific impact of myotonia. Consequently, myotonia management may be insufficient [15–17], despite symptomatic antimyotonic treatments having been investigated [10, 17–20]. A study using video-hand opening time to measure myotonia indicated that people with DM who have more-pronounced myotonia may have overall greater disease burden and worse quality-of-life (QoL) perception than those with less-extensive myotonia [21]. More data are required to support this finding.

Myotonia can be burdensome in DM [1, 2, 10, 22–24], but patients may find it hard to confirm which effects are directly myotonia related and which are due to ageing, fatigue, pain, or weakness, or even to dexterity problems relating to impaired executive function [1, 2, 12]. To gain a snapshot of real-world insight into myotonia, the ENSA (revEal the burdeN on daily life for myotonic dyStrophy patients due to myotoniA) online survey was undertaken.

## Methods

### Aims

ENSA was an international, cross-sectional, descriptive, anonymous, online survey conducted February 15–May 15, 2023, by admedicum^©^ (Cologne, Germany) for Lupin Neurosciences (Zug, Switzerland). Its primary aim was to explore the impact of myotonia in adults with DM; secondary goals were to investigate other physiological impacts of DM without focusing on cognitive impairments. Importantly, absence of myotonia was not an exclusion criterion. Antimyotonic treatment access was investigated, but information on specific pharmaceutical regimens was not sought, given the global differences in treatment availability.

## Population

### Ethical standards

The ENSA survey was approved in France by “Comité de protection des personnes Ouest III” (Committee for the Protection of Persons West III), ref: 22.04411.000149 and in the USA by Sterling IRB, ref: APP845, in accordance with ethical standards laid down in the 1964 Declaration of Helsinki and its amendments. Participants were informed of the survey’s objectives, risks and benefits of participation, confidentiality, sharing of results and their right to refuse or withdraw consent.

All participants provided informed consent before completing ENSA. The research was non-interventional, anonymous, and confidential: survey administrators had no direct contact with, and no means of identifying, participants.

### Participants

ENSA was targeted directly at adults aged ≥18 years with DM1 or DM2; if participants were aged <18, the survey automatically ended. Potential participants were informed about ENSA by routes including: direct communication from specialist neurologists or allied healthcare professionals in neuromuscular clinics; advertisements targeted to DM patient-advocacy groups (e.g. Myotonic Dystrophy UK, AFM-Téléthon, Deutsche Gesellschaft für Muskelkranke e.V., Myotonic Dystrophy Foundation, etc.); advertisements targeted at social media groups dedicated to DM; word-of-mouth via the DM community.

As ENSA was online-only, participants’ diagnoses could not be clinically verified at survey completion, but the survey asked if their DM was genetically confirmed. Nevertheless, lack of a genetic diagnosis was not an exclusion criterion. To allow for cognitive and fatigue-related burdens associated with DM, caregivers could help people to complete the survey, but ENSA sought patients’ (not caregivers’) views.

Although targeted at Canada, France, Germany, Italy, the Netherlands, Spain, the United Kingdom and the United States of America, people with DM living in any country could participate. The survey could be completed on hand-held devices (e.g. smartphones or tablets), or on PC/laptop platforms in English, French, German, Italian, Dutch or Spanish. The goal was to obtain 300 completed surveys.

### Development

The ENSA survey-development process and question flow are presented in Additional Files 1 A, B. In September 2022, an Advisory Board comprising clinical and academic myotonic dystrophy experts, a patient advocate, admedicum and Lupin Neurosciences representatives and a medical writer met to define and develop ENSA. The group agreed on participant eligibility (self-reported DM diagnosis) and the use of plain language to capture disease experiences and differentiate myotonia from other DM symptoms. Ultimately, ENSA comprised 32 questions on disease onset and diagnosis, symptom burden, daily activities, work/study, myotonia characteristics (frequency, location, impact, treatment), and patient priorities for improvement. The questionnaire was written in English for a lay audience, while retaining medical terminology commonly used in DM patient information. To support consistent interpretation, myotonia was defined as muscle stiffness, sudden seizing or delayed relaxation after contraction; muscle weakness was described separately for upper and lower body and explicitly distinguished from fatigue or myotonia. These definitions were applied consistently across the survey.

Questions were informed by established instruments, including the Individualized Neuromuscular Quality of Life Questionnaire [25] and EQ-5D-L [26], and used multiple-choice, free-text, Likert-scale [27], and Wong-Baker pain scale formats (Additional File 1B). Draft questions were reviewed iteratively by Advisory Board members and patient advocates. Cognitive and behavioural symptoms were excluded as they were outside the scope of ENSA. Given the exploratory nature of the study, the questionnaire was not pilot-tested prior to deployment.

### Translation and linguistic validation of the ENSA questionnaire

Following Advisory Board and patient-advocate approval of the English-language version, the questionnaire and associated recruitment materials were translated by admedicum and reviewed by native speakers to ensure linguistic clarity and cultural appropriateness. This process aimed to preserve conceptual equivalence across all versions in a manner appropriate for an exploratory survey. The revised survey was then implemented on the Survey Monkey online platform, where translations underwent additional review by native speakers to confirm accuracy and usability. Where feasible, further checks were performed by native-speaking collaborators, including patient advocates and clinical partners. In completed surveys, free-text responses submitted in languages other than English were translated into English and reviewed by native speakers to support consistent interpretation of qualitative data.

### Statistical analyses

Descriptive analyses were undertaken in cohorts with/without a self-reported genetically confirmed diagnosis, stratified by country, declared DM subtype, sex and age. Data are presented as N, with % or mean (range), and median (interquartile range; IQR or median (IQR) paired [distribution of differences across patients]) where relevant. Statistical analyses were conducted using SPSS Statistics version 28.0 (IBM, Chicago, IL, USA) and R [28], including sjPlot [29] and flextable [30]. Cronbach’s α with 95% confidence intervals was calculated to demonstrate the level of internal consistency reliability for assessment of the specific impact of myotonia on daily activities: psych package in R Software with pairwise deletion was utilised to maximise use of available item‑level data. Free-text answers are summarised narratively.

## Results

### Demographics

In total, 386 respondents aged 18–82 years completed ENSA. The majority came from the eight target countries (*n=*354; 91.7%; Additional File 2). *N=*238 (61.7%) were female and *n=*316 (81.8%) had DM1. *N=*70 (18.1%) questionnaires were submitted by caregivers.

### DM onset/diagnosis

**Fig. 1:**
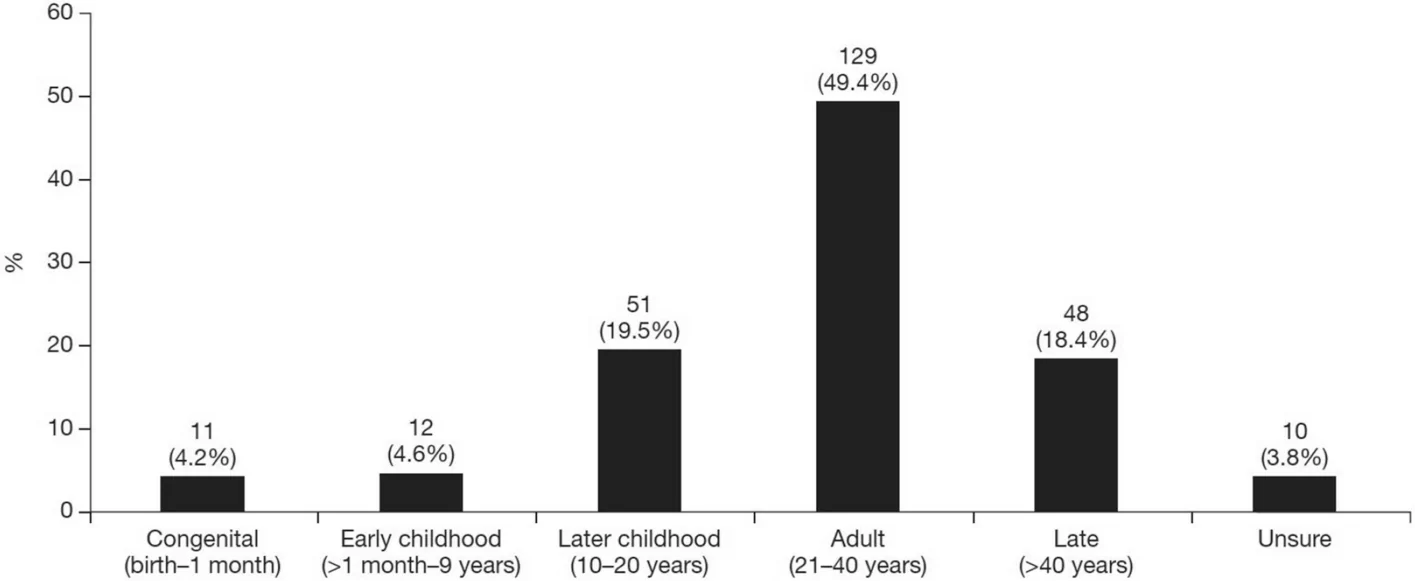
Proportions of different myotonic dystrophy (DM) type 1 subtypes declared by ENSA respondents who self-reported a genetically confirmed diagnosis (*n=*261)

Most respondents (354/386; 91.7%) self-reported a genetically confirmed diagnosis. DM1 was reported by *n=*261/354 (73.7%) (Fig. 1), including *n=*154/354 (59.0%) female respondents. *N=*93 (26.3%) people had DM2, of whom *n=*67 (72.0%) were female. Findings did not differ between the full cohort and the self-reported genetically confirmed cohort. Consequently, data for the self-reported genetically confirmed cohort are provided, unless otherwise stated, simply to give a slightly higher degree of confidence in participants’ diagnoses.

Of the 70 caregiver-completed responses, *n=*61 were for people with DM1 and *n=*9 were for people with DM2. Diagnostic information for the DM1 subcategories were: congenital *n=*2; childhood pre-puberty *n=*3; childhood late-puberty *n=*3; adult-onset age 21–40 *n=*21; adult late-onset age >40 *n=*11; unsure *n=*21.

### Symptom frequency

Although ENSA was a cross-sectional survey, symptom frequency was explored, including triggers to seek clinical support. Common symptoms/events to prompt a first clinical visit were often attributable to “myotonia” (*n=*113 [43%] respondents with DM1; *n=*43 [46%] with DM2). For DM2, first clinical visits were also prompted by “muscle weakness, lower body” (*n=*58; 54%) or “muscle ache/pain” (*n=*35; 38%).

**Figure 2:**
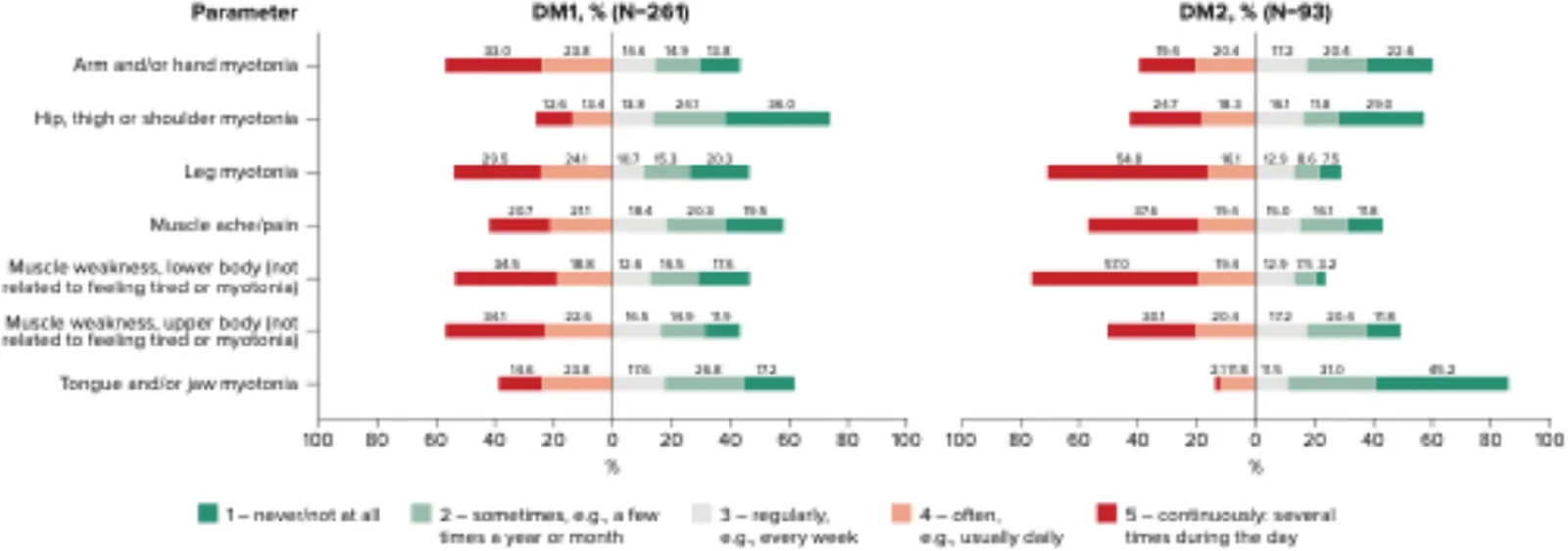
Heatmap graph of current neuromuscular symptom distribution/frequency in ENSA respondents. As expected, hand/arm myotonia was frequently described by people with myotonic dystrophy [DM] type 1. Leg myotonia was more prevalent in participants with DM2 than with DM1

Figure 2 shows the distribution and frequency of key neuromuscular symptoms. Myotonia and muscle weakness were widespread: only 5.4% of respondents with DM1 and 10.8% with DM2 had never experienced myotonia. On average, respondents experienced muscle ache/pain, hypersomnia, fatigue, leg myotonia and lower-/upper-body muscle weakness at least weekly. Symptom distribution and frequency are fully reported in Additional File 3.

### Impact on daily activities

**Fig. 3:**
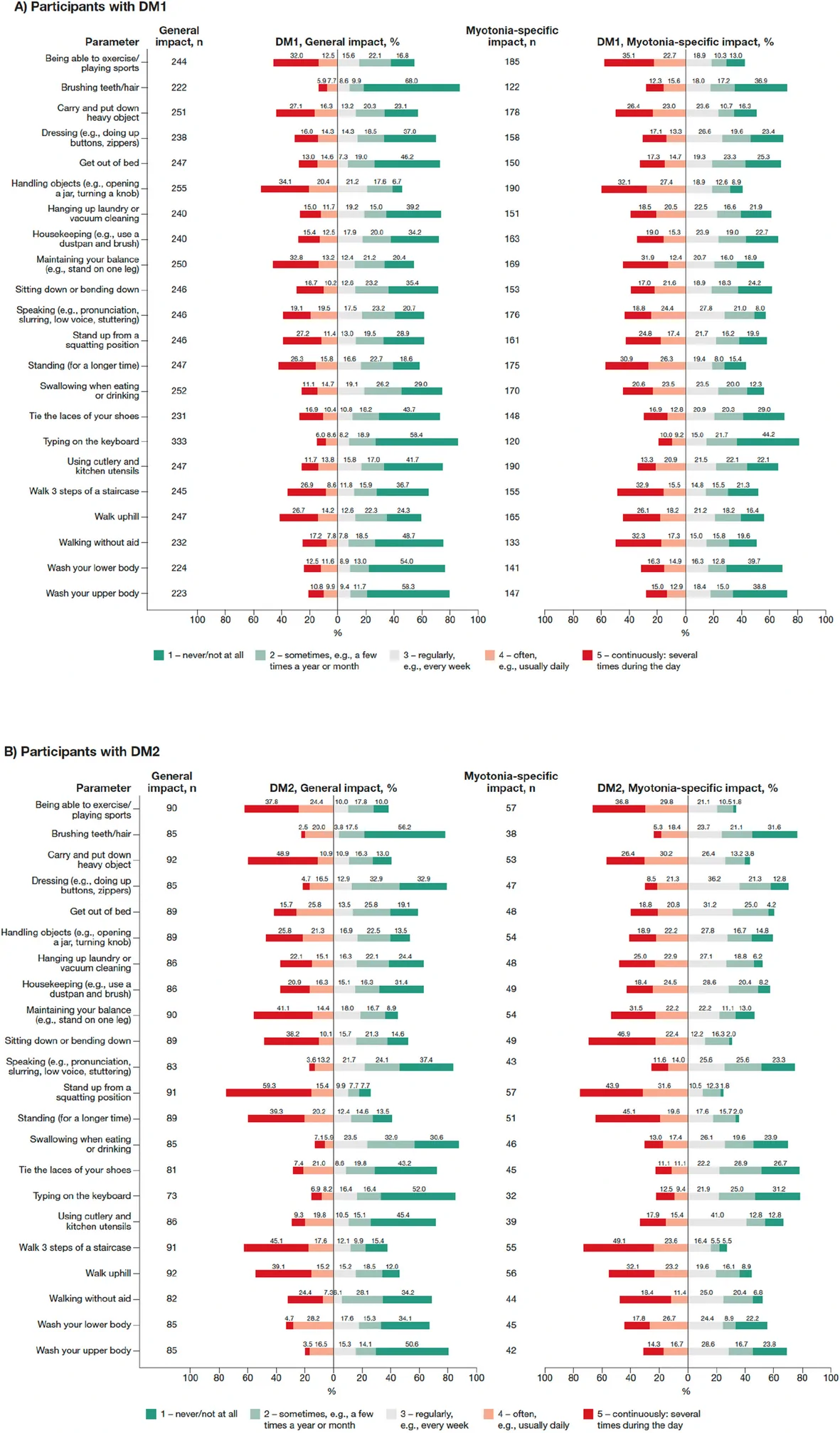
General (left) and myotonia-specific (right) impact of myotonic dystrophy (DM) on daily activities. **A** DM1 (*n=*261): 73% reported a myotonia-specific impact in at least one daily activity. Specific impact/burden of myotonia in DM1 was evident across most activities, indicated by the left shift of many responses in the right-hand graph. **B** DM2 (*N=*93): approximately half the cohort reported myotonia-specific impacts, but overall burden of disease had the greatest burden on respondents’ daily lives

General impact of DM and specific impact of myotonia are presented for DM1 (3A) and DM2 (Fig. 3B). Cronbach’s α was calculated to assess internal consistency reliability for items captured in Fig. 3, right-hand panels (i.e., myotonia-specific impact on daily activities), removing those with different intent (e.g., swallowing, speaking, typing). This represented a conceptually homogeneous set of items reflecting functional impact due to myotonia, and included 19 questions. Cronbach’s α were 0.96 (95% CI: 0.95–0.97) and 0.93 (95% CI: 0.91–0.95) for DM1 and DM2, respectively, indicating a highly coherent set of questions.

Although myotonia did not affect every respondent, the symptom negatively impacted exercise/sport, daily physical activities and swallowing (Additional File 4 A, B). Living with DM2 had a relatively high negative effect across most parameters.

**Fig. 4:**
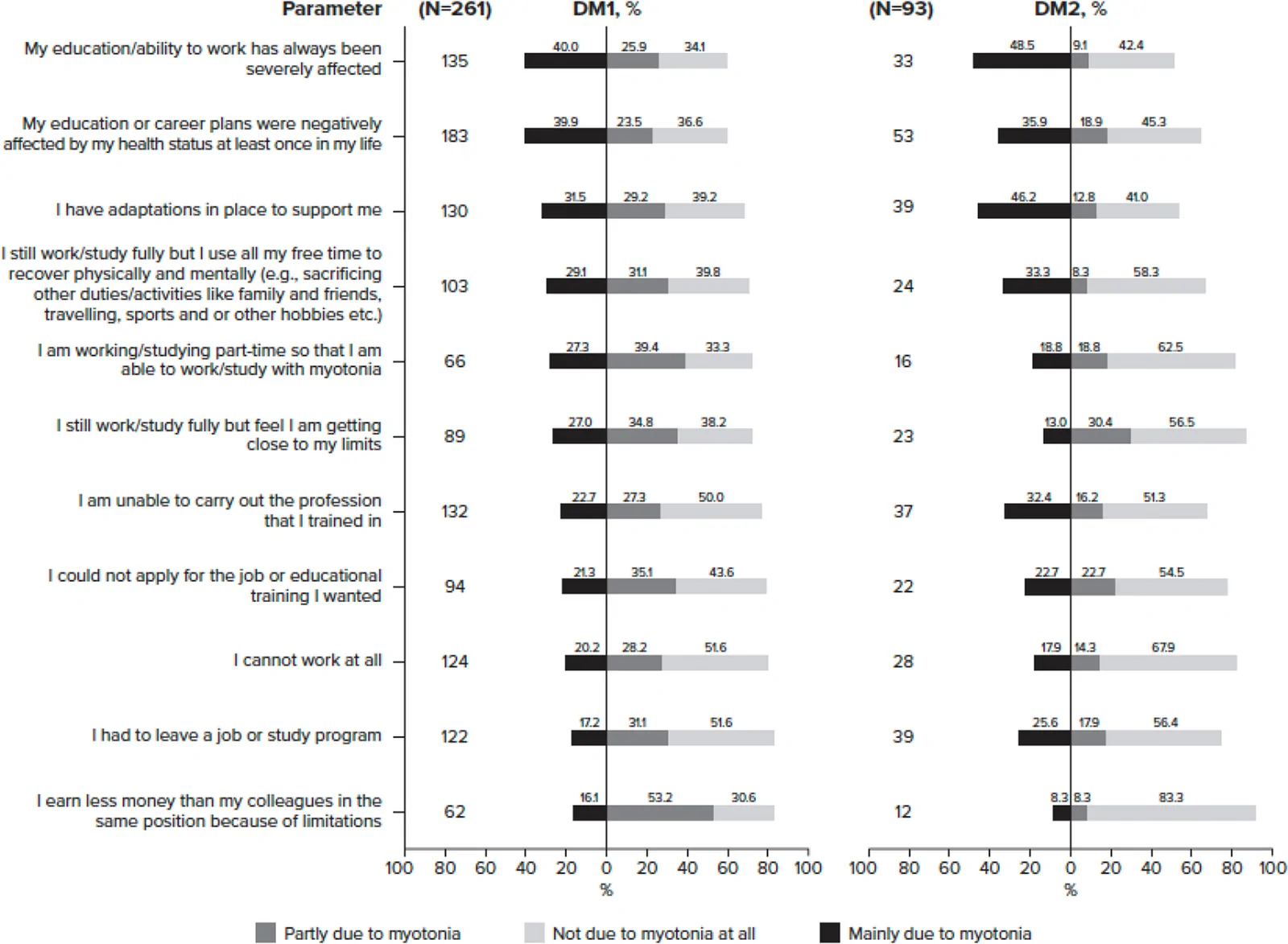
Heatmap to illustrate the direct impact of myotonia on work/study reported by ENSA respondents with myotonic dystrophy [DM] types 1 or 2

In free-text information (data not shown), *n=*16 respondents referred to “myotonia” as impacting or responsible for factors including: neck-muscle weakness/stiffness; dysphagia (associated with fear of choking, solid-food avoidance and surgical intervention to dislodge food); foot “contractures”; nocturnal cramping with ankle myotonia; problems keeping the mouth open while undergoing dental procedures). Hip and pelvic-girdle myotonia, excessive saliva, and unpredictability of DM symptoms were also considered myotonia-related. Myotonia negatively affected how respondents used the bathroom, exercised, undertook chores inside/outside the home, drove, shopped, performed repetitive movements (e.g. brushing hair), swallowed, ate, bent over, slept, walked long distances and dressed. Some people said that myotonia affected activities that are not clinically associated with myotonic symptoms (e.g., nerve pain or foot drop). Although myotonia was perceived as a symptom that could directly impact work or study (Fig. 4), people frequently had adaptations in place to support these activities.

### Age-related findings

For DM1 and DM2, respectively, median (IQR) ages were 25 (16–35) and 30 (23–45) years at symptom onset, 30 (22–40) and 45 (37–54) years at diagnosis, and 45 (37–53) and 55 (46–62) years at the time of completing ENSA. When interpreting the following results, it is important to remember that ENSA was cross-sectional: age-related findings do not reflect progression over time within the cohort.

Across the cohort (*n=*386), the median (IQR) age was 48 (38–58 years), and DM symptom frequency appeared to change with rising age. For example, reports of lower-body weakness increased with rising ages as expected (mean Likert scale scores; range 1 [never]–5 [continuous]), 2.7 in <40-year, 3.7 in ≥40 to <60-year, and 3.6 in ≥60-year age-groups) for DM1. For DM2, equivalent scores were 3.1 in <40-year, 4.2 in ≥40 to <60-year, and 4.4 in ≥60-year age-groups.

Leg myotonia was frequently reported by older respondents: mean Likert scale scores were 2.9 (DM1) and 3.7 (DM2) in the <40-year age-groups vs 3.6 (DM1) and 3.1 (DM2) for ≥40 to <60-year age-groups. Reports of balance problems/falls increased with respondents’ rising age (mean Likert scores peaking at 3.4 for both cohorts in ≥60-year age-groups). In DM1, arm/hand myotonia was experienced weekly: the mean Likert score was 3.5 across age-groups. In DM2, mean arm/hand myotonia scores were higher in the <40-year age-group (3.6) than the cohort average score (2.9).

### Myotonia management

Pharmacological treatment rates were low: 54/247 (21.9%) respondents with DM1 and 26/83 (31.3%) with DM2 were on antimyotonic treatment; 149/247 (60.3%) respondents with DM1 and 40/83 (48.2%) with DM2 had never received an antimyotonic. Few respondents were satisfied (59/354; 16.7%) or very satisfied (16/354; 4.5%) with myotonia management.

### Patients’ expectations

As per the question flow (Additional File 1B), respondents were asked which symptoms were most important to them before they were asked about personal impact of myotonia, or treatment experiences/expectations. Responses were grouped by those who were, or were not, receiving antimyotonic treatment.

Respondents with DM1 who were already on an antimyotonic most wanted to improve muscle weakness (39/54; 72.2%), fatigue (27/54; 50%), myotonia (27/54; 50%), hypersomnia (18/54; 33.3%), muscle ache/pain (18/54; 33.3%), or balance problems/falls (17/54; 31.5%). Those not on an antimyotonic most wanted to improve muscle weakness (134/193; 69.4%), fatigue (111/193; 57.5%), myotonia (104/193; 53.9%), hypersomnia (80/193; 41.5%), or balance problems/falls (74/193; 38.3%) (Additional File 5).

Respondents with DM2 on antimyotonic treatment most wanted to improve muscle weakness, muscle ache/pain or fatigue (all 18/26; 69.2%), balance problems/falls (12/26; 46.2%), or myotonia (11/26; 42.3%). Those not on an antimyotonic most wanted to improve muscle weakness (48/57; 84.2%), muscle ache/pain (30/57; 52.6%), fatigue (29/57; 50.9%), balance problems/falls (25/57; 43.9%), insomnia (22/57; 38.6%) or myotonia (19/57; 33.3%) (Additional File 5).

**Fig. 5:**
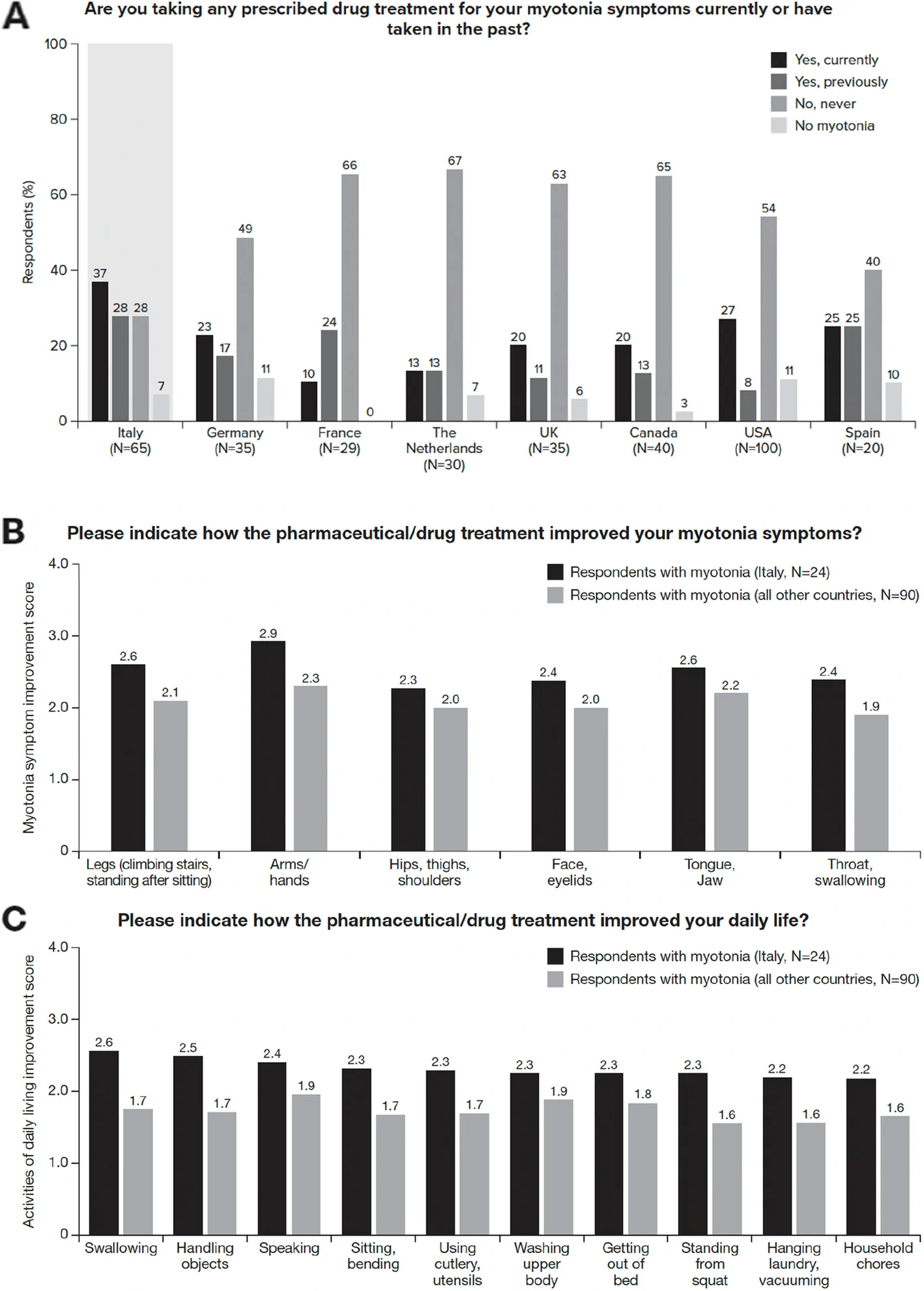
Differences in myotonia treatment and burden between the top eight countries contributing ≥20 respondents to ENSA (full cohort) conducted in people with myotonic dystrophy types 1 or 2. **A** Reported levels of myotonia treatment. Perceptions of treatment impact on **B** myotonia symptoms and **C** daily life. Improvements in symptoms and daily life were scored on Likert scales (4=improved significantly; 3=improved moderately; 2=improved occasionally; 1=did not improve at all. “Not applicable” responses excluded. Italian results are emphasised because of the high proportion of treatment use among this cohort

There were between-country differences in myotonia treatment, treatment satisfaction and burden (Fig. 5A–C). Respondents in Italy (*n=*65) had the highest rate of treatment (37%) and the lowest rate of no treatment (28%). They also had the highest treatment-related improvements in undertaking daily activities, and the lowest treatment dissatisfaction score (mean 24.6% for Italy vs. mean [range] 40.0% [31.4–55.0%] for other target countries).

### Additional findings

Median (IQR) paired diagnostic delays were 3 (0–10) years for DM1 and 12 (4–19) years for DM2. For DM1, median (IQR) paired diagnostic delays were 3 (0–9) years for people with myotonia at onset vs. 4 (0–14.5 years) for those with no myotonia at onset. For DM2, median (IQR) paired diagnostic delays were 11 (5–12.5) years for people with myotonia at onset vs. 14.5 (3.75–19.2) for those with no myotonia at onset. *N=*52 (13.5%) people with DM2 were diagnosed while asymptomatic, *n=*45 (86.5%) of whom had a self-reported genetically confirmed diagnosis: 44/45 (97.7%) had a family history of DM2.

When DM1/DM2 data were pooled, median (IQR) paired diagnostic delays were 4 (0–13) years for those with a self-reported genetic predisposition vs. 8 (2.5–13.5) years for those without a genetic predisposition, and 7 (2.0–15.2) years for those who did not know their genetic history.

No noteworthy sex-related differences in symptom burden, treatment exposure or treatment goals were observed (data not shown).

## Discussion

The ENSA questionnaire exceeded its target number of respondents, which is encouraging for a symptom-focused survey, given that apathy and limited disease awareness are common in DM [4, 5]. As an anonymous, online, cross-sectional survey open to adults with DM, ENSA provided a pragmatic snapshot of patient-reported symptom burden outside traditional clinical research settings and complemented data derived from clinic-based cohorts.

A key design feature of ENSA was the inclusion of respondents regardless of whether they perceived myotonia to be a prominent symptom. This approach reduced the risk of selecting only those who are particularly focused on myotonia, enabling interpretation of myotonia within the broader symptom framework of DM. Accordingly, ENSA findings align with previous studies showing that DM symptom burden is heterogeneous and multidomain, and that patient priorities extend beyond any single manifestation[2, 10, 11, 13, 14].

Beyond myotonia frequency and distribution, ENSA provides patient-reported context on how this core symptom of DM is experienced and interpreted in daily life. Many respondents described the negative impact of myotonia on routine tasks, health, wellbeing, education and career prospects. These findings are directionally consistent with emerging quantitative research using objective measures of myotonia, such as video-based hand opening time, in which more-pronounced myotonia was associated with heightened disability levels [21]. Together, these data corroborate that myotonia contributes meaningfully to the extent of personal functional limitations in DM, and highlight challenges that people face when separating specific effects of myotonia from those of weakness, fatigue, pain, or other multisystemic manifestations of DM.

When asked about priorities for improvement, ENSA respondents frequently selected muscle weakness, fatigue and myotonia irrespective of their declared DM subtype. This broadly concurs with data from the Myotonic Dystrophy Family Registry in which fatigue, myotonia, hypersomnia, pain and difficulty walking were often reported [12]. ENSA findings also align with DM1 data in which muscle weakness, fatigue and myotonia were significantly associated with high levels of both general and QoL burden [1, 2, 10, 31]. A QoL study noted that myotonia was less severe in people with adult-onset DM2 compared with DM1: QoL in DM2 was most affected by ageing, fatigue and poor muscle strength [32]. Again, ENSA data align with these findings.

ENSA also highlighted areas of potential patient–clinical ambiguity, worthy of future study. Some respondents attributed oropharyngeal (swallowing) difficulties as being myotonia-related, affecting their food choices for fear of choking. Approximately 70% of the DM1 cohort reported that experiencing myotonia “at least weekly” affected their ability to handle objects or exercise, detrimentally affecting walking, swallowing or speaking. Respondents’ accounts of myotonia during dental procedures were also noteworthy: DM is associated with temporomandibular joint dysfunction, and orthodontic evaluation may be necessary in affected patients [33, 34]. As expected, in ENSA the general impact of DM2 on standing, walking, exercising, carrying heavy objects and maintaining balance was high: DM2 typically affects lower limbs. However, the specific impact of myotonia reported by patients with DM2 was often considerable.

In ENSA, antimyotonic treatment was uncommon, which was expected because none is licensed, although several are used off label [20, 35]. However, only 22% of respondents with DM1 and 31% with DM2 were on an antimyotonic and there are many reasons for their limited use. For example, clinicians may reserve mexiletine for functionally impactful cases [36]. Cardiac safety may be a concern, although guidance is published to streamline cardiac assessment and monitoring requirements before and during mexiletine treatment [37]. In general, however, people with DM may have limited awareness of antimyotonic treatments [38] and how they may reduce disease burden [38, 39].

Notably, respondents on antimyotonic treatment reported similar desire to further improve myotonia compared with those not on treatment. This apparent lack of difference should be interpreted cautiously. ENSA was not designed to compare treatment effectiveness, and its initiation is likely influenced by baseline symptom severity. Patients on antimyotonics may therefore represent a subgroup with more severe or functionally impactful myotonia at baseline, which could explain the persistence of unmet need.

Variability in international drug access, dosing, tolerability and expectations may further contribute to the mixed findings regarding antimyotonic treatment seen in ENSA. For example, Italy has a proactive strategy for DM management and a relatively high level of antimyotonic use [18, 24]. ENSA respondents in Italy had positive perceptions of the impact of treatment on myotonia relief and improvements daily life, compared with others (Fig. 5 A-C). Nevertheless, strong conclusions about treatment expectations cannot be drawn from ENSA data. The findings in Italy likely reflect differences in healthcare pathways and treatment familiarity rather than differences in treatment efficacy. The international HERCULES study (and its open-label extension, ATLAS) is investigating the efficacy and safety of once-daily mexiletine prolonged release in people with DM and may provide more robust information on patient’s treatment expectations than could be provided in ENSA data [40].

We acknowledge potential limitations to the ENSA survey. Presently, no tools exist to provide objective assessment of neuromuscular function and strength in remotely conducted online surveys. Consequently, ENSA relied on patient‑reported perceptions, which may contribute to overlap or misattribution between myotonia and other symptoms, despite the repeated use of explanatory definitions to differentiate them. While we lack concrete evidence that such confusion occurred, we cannot fully exclude this possibility. We aimed to record patients’ perceptions of how myotonia contributes to overall daily burden within the broader disease context, rather than to assign functional impairments exclusively to a single mechanism. Importantly, symptom overlap does not negate myotonia’s burden in DM; rather, it highlights that patients experience disease holistically.

In DM, muscle weakness, fatigue or pain may be independently present or may occur because of myotonia. Studies report blurring of experiences involving different symptoms (such as myotonia, muscle weakness, central-nervous system effects and fatigue) [1, 2, 13]. Some ENSA respondents associated myotonia with foot drop and neuralgia, when weakness or pain may have been causative. In addition, the specific impact of myotonia on study or work may not have been clearly demonstrated by ENSA data. Certainly, myotonia directly affected some respondents (e.g. limiting the ability of a nurse to practice, or manual workers to use tools), but other free-text answers were not myotonia relevant.

Although caregiver-completed responses may not fully capture subjective aspects of myotonia in cognitively impaired individuals, we believe this limitation does not materially affect the present analyses, given the predominance of adult-onset DM1 respondents and the study’s descriptive, non-subtype-specific objectives. ENSA could be adapted for specific populations, including people with congenital or even childhood-onset DM1, in future.

Our limited reliability testing only involved multi-item domains relating to impact of myotonia on daily activities. Although testing showed very high internal consistency, reflecting the expected co-occurrence of functional impacts on daily life, given the cross-sectional, anonymous nature of ENSA, test–retest reliability could not be performed. In addition, our questionnaire did not undergo formal pilot validation of its structure, accessibility or readability. No exploratory factor analysis was conducted, as the primary focus of ENSA’s development phase was on questionnaire creation and initial application. Likewise, correlations with established instruments including the Myotonic Dystrophy Health Index (MDHI, favoured by study participants with DM1 [41]), or SF‑36 were not performed. Examining whether myotonia‑specific impacts are clearly distinguishable from overall disease burden**,** and analysing criterion validity through correlations with relevant subscales (e.g., the myotonia domain of the MDHI) will be important for ENSA. In addition, symptom worsening was not included as a Likert scale option: we deemed it to be covered by “did not improve at all”, but for clarity it could be added.

Our cross-sectional investigations focused on symptomatology, not DM subtype, diagnostic odyssey or disease duration. DM onset may not be clear, often because of its multisystemic nature and poor public awareness [4, 5], but we believe that our initial findings provide real-world evidence of the ongoing, substantial, delay between perceived onset and diagnosis, and potential areas of clinical confusion facing patients with DM. Also, we did not collect data on the number of CTG repeat expansions. To retrieve and input such information could have discouraged spontaneous survey completion. However, this omission prevented us from conducting genotype–phenotype correlations or obtaining additional diagnostic confirmation. In addition, we acknowledge that the proportions of patients with certain DM subtypes may be biased, given that there were no limitations on including certain forms of DM.

To conclude, ENSA findings show that neuromotor function is impaired in DM1, provide useful insight into the personal burden of myotonia on people with DM1 or DM2, and indicate that individuals affected by myotonia may value symptomatic treatment. The low reported use of antimyotonic treatment among ENSA respondents, despite the perceived myotonia burden, suggests ongoing unmet needs. Further work is needed to understand barriers to symptomatic myotonia management, refine patient-reported assessments, and evaluate treatment effects in people with DM. Such work includes appropriately designed clinical studies such as HERCULES, which is in progress.

## Supplementary Information

Supplementary Material 1.

## Data Availability

Datasets used and/or analysed during the current study are available from the corresponding author on reasonable request.

## Abbreviations

DM: Myotonic dystrophy
IQR: Interquartile range
QoL: Quality of life
