# Supplementary material for: Exploring the impact of myotonia on daily functioning in myotonic dystrophy: a patient-reported survey
Source: BMC Neurol. 2026 Jun 16;26:538. doi: 10.1186/s12883-026-05002-4 (PMC13501778; doi:10.1186/s12883-026-05002-4)
Supplement: Supplementary file 1 [file 12883_2026_5002_MOESM1_ESM.pdf]

Additional File 1. (A) Graphical representation of ENSA survey development process; **(B)** questionnaire flow: questions were programmed into an online form.

A

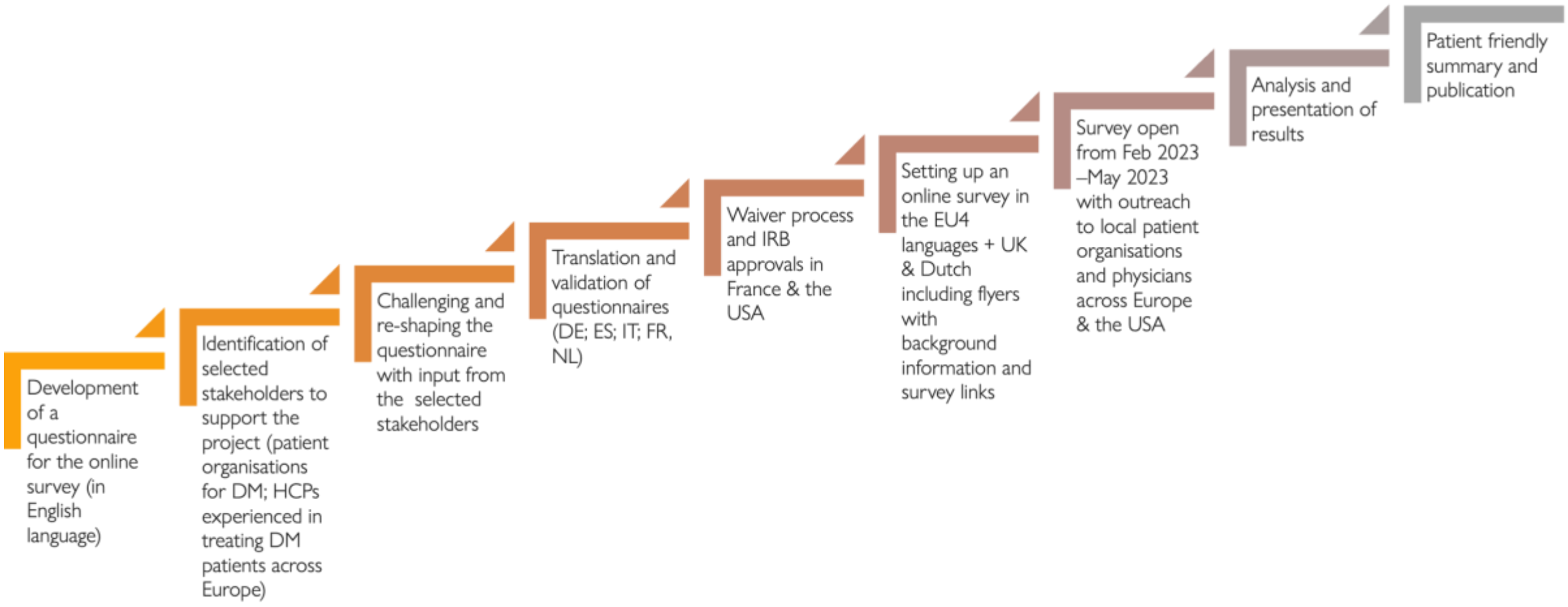

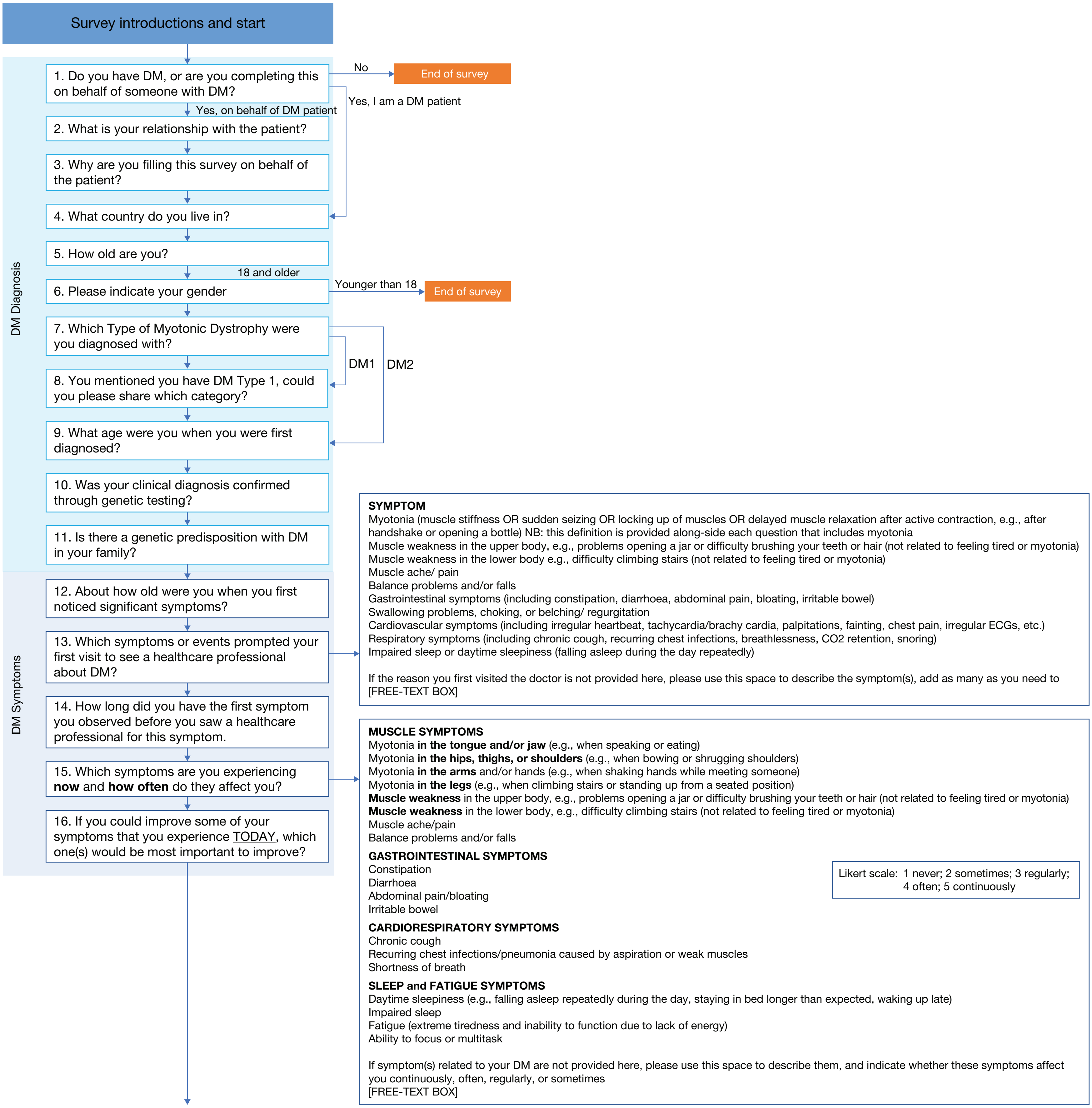

Survey introductions and start
DM Diagnosis
1. Do you have DM, or are you completing this on behalf of someone with DM?
No
End of survey
Yes, I am a DM patient
Yes, on behalf of DM patient
2. What is your relationship with the patient?
3. Why are you filling this survey on behalf of the patient?
4. What country do you live in?
5. How old are you?
18 and older
Younger than 18
End of survey
6. Please indicate your gender
7. Which Type of Myotonic Dystrophy were you diagnosed with?
DM1
DM2
8. You mentioned you have DM Type 1, could you please share which category?
9. What age were you when you were first diagnosed?
10. Was your clinical diagnosis confirmed through genetic testing?
11. Is there a genetic predisposition with DM in your family?
DM Symptoms
12. About how old were you when you first noticed significant symptoms?
13. Which symptoms or events prompted your first visit to see a healthcare professional about DM?
14. How long did you have the first symptom you observed before you saw a healthcare professional for this symptom.
15. Which symptoms are you experiencing **now** and **how often** do they affect you?
16. If you could improve some of your symptoms that you experience TODAY, which one(s) would be most important to improve?
SYMPTOM
Myotonia (muscle stiffness OR sudden seizing OR locking up of muscles OR delayed muscle relaxation after active contraction, e.g., after handshake or opening a bottle) NB: this definition is provided along-side each question that includes myotonia
Muscle weakness in the upper body, e.g., problems opening a jar or difficulty brushing your teeth or hair (not related to feeling tired or myotonia)
Muscle weakness in the lower body e.g., difficulty climbing stairs (not related to feeling tired or myotonia)
Muscle ache/ pain
Balance problems and/or falls
Gastrointestinal symptoms (including constipation, diarrhoea, abdominal pain, bloating, irritable bowel)
Swallowing problems, choking, or belching/ regurgitation
Cardiovascular symptoms (including irregular heartbeat, tachycardia/brachy cardia, palpitations, fainting, chest pain, irregular ECGs, etc.)
Respiratory symptoms (including chronic cough, recurring chest infections, breathlessness, CO2 retention, snoring)
Impaired sleep or daytime sleepiness (falling asleep during the day repeatedly)
If the reason you first visited the doctor is not provided here, please use this space to describe the symptom(s), add as many as you need to [FREE-TEXT BOX]
MUSCLE SYMPTOMS
Myotonia **in the tongue and/or jaw** (e.g., when speaking or eating)
Myotonia **in the hips, thighs, or shoulders** (e.g., when bowing or shrugging shoulders)
Myotonia **in the arms** and/or hands (e.g., when shaking hands while meeting someone)
Myotonia **in the legs** (e.g., when climbing stairs or standing up from a seated position)
Muscle weakness in the upper body, e.g., problems opening a jar or difficulty brushing your teeth or hair (not related to feeling tired or myotonia)
Muscle weakness in the lower body, e.g., difficulty climbing stairs (not related to feeling tired or myotonia)
Muscle ache/pain
Balance problems and/or falls
GASTROINTESTINAL SYMPTOMS
Constipation
Diarrhoea
Abdominal pain/bloating
Irritable bowel
Likert scale: 1 never; 2 sometimes; 3 regularly; 4 often; 5 continuously
CARDIORESPIRATORY SYMPTOMS
Chronic cough
Recurring chest infections/pneumonia caused by aspiration or weak muscles
Shortness of breath
SLEEP and FATIGUE SYMPTOMS
Daytime sleepiness (e.g., falling asleep repeatedly during the day, staying in bed longer than expected, waking up late)
Impaired sleep
Fatigue (extreme tiredness and inability to function due to lack of energy)
Ability to focus or multitask
If symptom(s) related to your DM are not provided here, please use this space to describe them, and indicate whether these symptoms affect you continuously, often, regularly, or sometimes
[FREE-TEXT BOX]

Managing Daily life with DM

17. Please indicate how often you struggle with the following activities due to DM 1 or DM

18. Please rate now how important it would be for you to improve or to be able to do these activities:

PERSONAL NEEDS

Likert scale: 1 never; 2 sometimes; 3 regularly; 4 often; 5 continuously

- Wash your upper body (e.g., hair and shoulders)
- Wash your lower body (e.g., feet)
- Brushing teeth/ hair
- Dressing (e.g., doing up buttons, zippers)
- Tie the laces of your shoes
- Speaking (e.g., pronunciation, slurring, low voice, stuttering)
- Get out of bed
- Walking without aid
- Stand up from squatting position
- Sitting down or bending down
- Walk uphill
- Walk 3 steps of a staircase
- Standing (for a longer time)
- Maintaining your balance (e.g., stand on one leg)
- Carry and put down heavy object (e.g., lifting groceries, move a chair)
- Typing on the keyboard
- Being able to exercise/playing sports (e.g., working in the garden, run or catch an object/ a ball)
- Housekeeping (e.g., use a dustpan and brush)
- Hanging up laundry or vacuum cleaning
- Handling objects (e.g., opening a jar, turning knob)
- Using cutlery and kitchen utensils (e.g., cutting things)
- Swallowing by eating or drinking (e.g., eating soup)

Work and Study

19. We would like to learn more about how **DM** in general has affected your ability to work or study.

Life aspect

Choose all that apply

- I have little or no restrictions on my working life/study
- The disease negatively affected my education or career plans at least once in my life
- I have adaptations in place to support me
- I still work/study fully but I use all my free time to recover physically and mentally (e.g., sacrificing other duties/activities like family and friends, travelling, sports and or other hobbies)
- I still work/study fully but feel I am getting close to my limits
- I am working/studying part-time so that I am able to work/study with DM
- My education/ability to work has always been severely affected
- I could not apply for the job or educational training I wanted
- I had to leave a job or study program
- I am unable to carry out the profession that I trained in
- I earn less money than my colleagues in the same position because of limitations
- I cannot work at all
- Other, please specify: [FREE-TEXT BOX]

Managing Daily Life with Myotonia

20. Have you **EVER** (currently or in the past) experienced symptoms of myotonia (muscle stiffness OR sudden seizing OR locking up of muscles OR delayed muscle relaxation after active contraction, e.g., after handshake or opening a bottle)? This could be anywhere in your body, for example your arms, hands, legs, face, mouth/tongue, or eyelids.

No → 32; Yes, in the past → 23; Yes, today → 21

Myotonia experienced today

21. Please provide more specific information about where/how often myotonia affects you **CURRENTLY**

22. Please rate how much **Myotonia affects the ability to do the following activities CURRENTLY**

MYOTONIA SYMPTOMS

- Myotonia **in the legs** (e.g., when climbing stairs or standing up from a seated position)
- Myotonia **in the arms and/or hands** (e.g., when shaking hands while meeting someone)
- Myotonia **in the hips, thighs or shoulders** (e.g., when bowing or shrugging shoulders)
- Myotonia **in the face** (e.g., problems opening eyes after prolonged contraction)
- Myotonia **in the tongue and/ jaw** (e.g., when chewing or biting)
- Myotonia **in the throat** (e.g., when swallowing)
- Other, please specify: [FREE-TEXT BOX]

See Footnote

23. How old were you when you first started experiencing myotonia?

24. Are you taking any drug treatment for your myotonia symptoms today or have taken it in the past?

No drug treatment at all → 29; Taken in the past → 28; Today → 25

Drug treatment today

25. Please provide more specific information about where and how often myotonia affected you before you started drug treatment.

26. Please indicate how the pharmaceutical/drug treatment improved your myotonia symptoms.

27. Please indicate how the pharmaceutical/drug treatment improved your daily life.

Drug treatment in the past

28. What are the reasons you stopped receiving medical treatment for myotonia?

Please click all that apply

- My stiffness/myotonia got much better and I decided to stop
- I didn't want to take any drugs anymore, it was my personal decision
- My doctor could not prescribe the medicine any more
- I had to travel too far or too frequently to get a prescription
- The process to obtain the treatment was too burdensome
- The medicine was too expensive
- I am not sure why I stopped the treatment
- Other, please specify: [FREE-TEXT BOX]

29. You shared that you never received medical treatment for myotonia, what are the reasons for this?

Please click all that apply

- My doctor never offered a medical drug treatment to manage myotonia
- I'm concerned about side effects of drug treatment
- I'm concerned that my health will get worse
- My myotonia/muscle stiffness symptoms are managed well without any drug therapy
- I do not want to take any drug treatment
- I have never heard of any drug treatments for myotonia/muscle stiffness
- The administrative effort/ paperwork required to obtain medication is inconvenient for me and /or my doctor
- The medicine is too expensive
- All myotonia treatments are off label and my doctor would not prescribe any drugs that are not explicitly approved for the treatment of myotonia
- I have myotonia but it does not bother me
- Other, please specify: [FREE-TEXT BOX]

30. Are you satisfied how your myotonia is managed today?

Work and Study

31. How much of the impact or restriction on your work/study was a direct **result of myotonia**:

Life aspect

- Myotonia negatively affected my education or career plans at least once in my life
- I have adaptations in place to support me
- I still work/study fully but I use all my free time to recover physically and mentally (e.g., sacrificing other duties/activities like family and friends, travelling, sports and or other hobbies etc.)
- I still work/study fully but feel I am getting close to my limits
- I am working/studying part-time so that I am able to work/study with myotonia
- My education/ability to work has always been severely affected
- I could not apply for the job or educational training I wanted
- I had to leave a job or study program
- I am unable to carry out the profession that I trained in
- I earn less money than my colleagues in the same position because of limitations
- I cannot work at all
- Other, please specify: [FREE-TEXT BOX]

32. Finally, is there anything else that you would like to mention about your experiences with DM that has not already been covered in the survey?

End of survey

Footnote

Questions followed Likert scale (e.g., range 1–5: 1=Never; 5=Continuously [several times daily] or 1–4: 1=Did not improve at all;4=Improved significantly) and Wong–Baker pain facial scale formats. Where necessary, participants could expand answers in free-text boxes

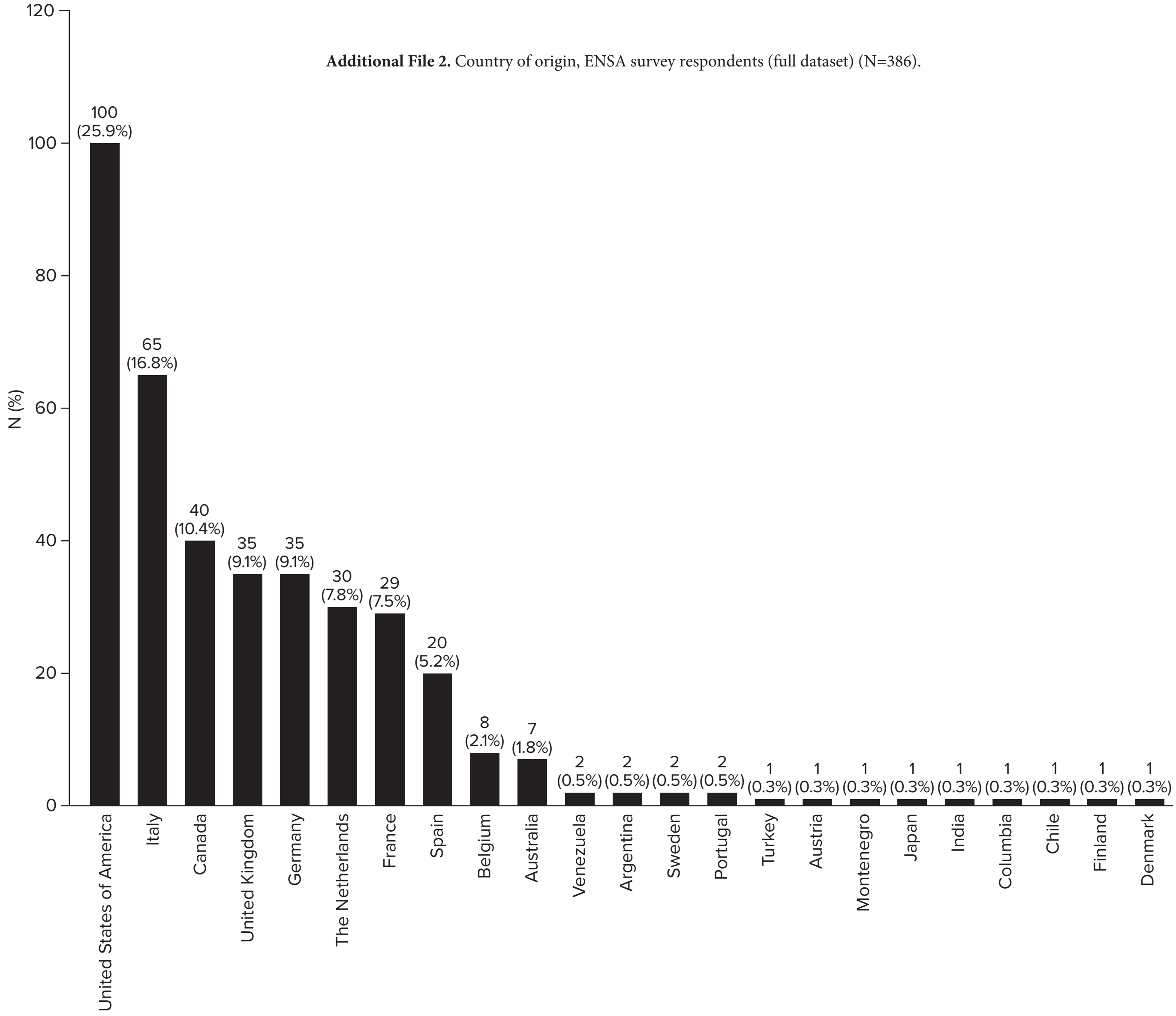

Additional File 2. Country of origin, ENSA survey respondents (full dataset) (N=386).

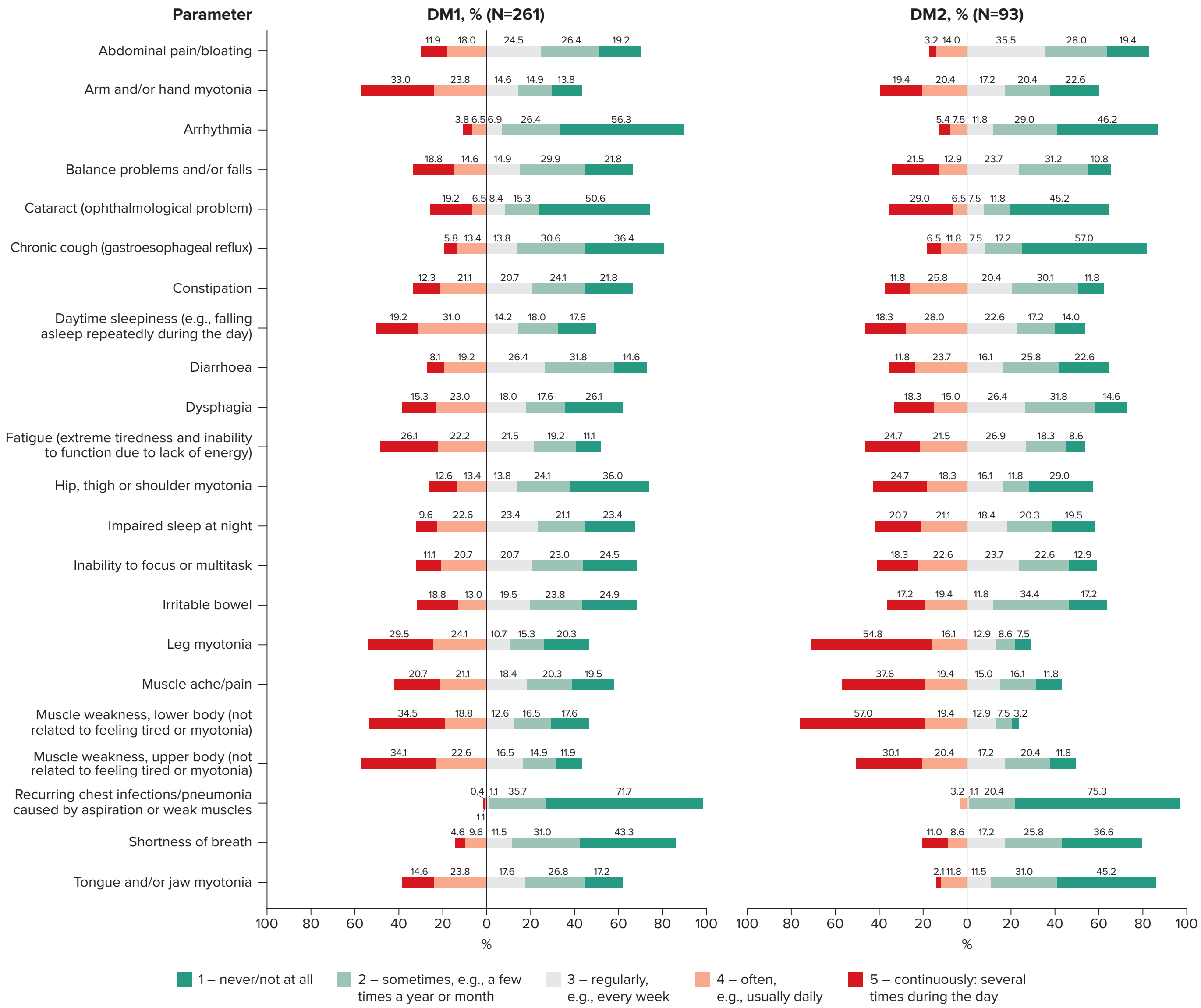

Additional File 3. Heatmap graph showing full symptom distribution/frequency at the time of the ENSA survey (expansion of Figure 2 in main text).

Additional File 4. (A) Free-text information provided by respondents to describe the impact of myotonia on their daily ability to undertake activities

Free text information
Again, I can do sports, I can do household chores, carry heavy objects - I think it's just more of a struggle or more tiring than the average person.
I avoid exercise, but household chores I can't really avoid.
Going to the bathroom, wiping, driving.
Walking without drooping feet.
Driving, especially in the evening.
Can't stand on steps to clean windows properly. Have to hold onto something if lifting head up to deal with (cobwebs, etc.).
It's hard for me to get around with foot drop, so I have given up driving and shopping.
Repetitive movements, such as brushing my daughter's hair, have a significant impact.
My swallowing difficulties occur only occasionally.
A lot.
There is much focus on household chores. I feel you neglected outdoor activities, taking care of the house, for example, house, yard, and vehicle maintenance. Things my husband used to do that he has trouble doing now, such as shovelling snow, building a shed, taking our docks in and out of the water in spring and fall.
Needs constant assistance with eating!
Bending over in a seated position.
Sleeping. Myotonia in hips and low back after more than 4 hours of sleep at a time.
Putting on pants and underwear is severely difficult.
Tense muscles and muscle atrophy cause pain and nerve pain. I work out my lower body but can't do much for the upper body to maintain strength due to pain and muscle tensing. This will cause me more issues in the future.
Walking long distances.

Additional File 4. (B) Summary of free-text responses to the question "How much of the impact or restriction on your work/study was a direct result of myotonia?"

**Respondent considered impact of DM to be multifactorial, not (or not only) due to myotonia**
Cannot work due to many factors of my DM, lack of strength being one. I have no idea how myotonia is the factor. I have no idea which are due to myotonia, so I can't answer
Muscle weakness, poor breathing. Wheelchair-bound due to muscle weakness, heart failure (bundle branch, low EF, AF, many other issues)
Muscle pain and extreme muscle fatigue are the inhibiting factors for me, more so than myotonia itself
The pain and weakness are my biggest concerns and affect everything in my life
Muscle weakness is worse than the myotonia
Also because of heart transplant
Chair-bound and on a ventilator
Due to constant weakness, fatigue, and pain, severe depression has also occurred
Everything is affected by my DM1, but not myotonia directly
Fatigue is a big part of my issues as well
Fatigue
Fatigue, cognitive, and emotional impacts
I am unable to work due to fatigue and muscle weakness, not so much from myotonia
Mainly because of DM1, which affects my whole body to some extent
I am a caregiver for my daughter. I was made redundant years ago and didn't take up work as I was pregnant, severely tired and then had a daughter with CDM
I currently work, and my myotonia has only happened one time, so it does not impact me in any way at all
Also the death of my daughter
**Respondent made a direct connection between myotonia and with employment/health insurance issues**
Incapacity
Unable to work
No adaptation implemented by my supervisor
I can only work from home
Early retirement due to illness (x 5)
I retired early because I started having problems handling the tools of my trade
I felt I had to retire before I was due to do so because my manual dexterity was so poor

I work reduced hours at my main job. I think I will be forced to retire [from volunteer first responder role]
While I'm on medically forced retirement and have difficulty driving with fatigue, I do consult
I worked as an nurse. Now on long-term disability as my myotonia, fatigue, and stamina have impacted my job
I actually wanted to learn a craft-creative profession. Even back then, due to my heart condition, visual impairment, and cataracts, I was advised against it. As mentioned, I did not know about my condition at that time. I want to switch to working from home, which is relatively difficult due to the tasks involved. [The data-input job I do is changing], so it's hardly feasible. I am grateful that I still have a job because I couldn't take much care of my health. Insurance companies have always rejected me, and even more so now. The disability pension is so little that I wouldn't be able to manage as a single person
Insufficient coverage for physiotherapy
I was self-employed, can no longer work, no disability pension
Was told to leave work and go on long-term disability due to MD - kept falling asleep
While my employment is not affected now, I am concerned that as my speech becomes worse, it will have a negative impact on future employment opportunities
**Respondent associated myotonia with aging** ***(no further explanations given)***
Retired (x 4)
I am retired and feel I am restricted in most things
Age
**DM and myotonia are problematic but respondent tries to overcome hurdles**
I work a lot. I'm on the move all day. I am the owner of a tavern. I think all myotonia symptoms would be worse if I had more time to think about it
I work full time and own my own business. I am actively following all the trials for MD and CRISPR in hope that we can reverse the genetic damage
I am self-employed, I have employees, I can manage my own time, although I sacrifice free time to dedicate more hours to work and advance my profession and business

Additional File 5. Symptoms that respondents to ENSA wished to improve. Patients were presented with common symptoms (described using lay language; pain and myotonia were differentiated) and free-text space in which to list other symptoms they experienced, but are grouped below using medical terminology.

Symptom respondent wishes to improve	DM1, N (%)		DM2, N (%)	
	On myotonia treatment	Not on myotonia treatment	Receiving myotonia treatment	Not receiving myotonia treatment
Muscle weakness (anywhere in body)	39 (72.2)	134 (69.4)	18 (69.2)	48 (84.2)
Fatigue	27 (50.0)	111 (57.5)	18 (69.2)	29 (50.9)
Myotonia (anywhere in body)	27 (50.0)	104 (53.9)	11 (42.3)	19 (33.3)
Hypersomnia	18 (33.3)	80 (41.5)	5 (19.2)	13 (22.8)
Balance problems and/or falls	17 (31.5)	74 (38.3)	12 (46.2)	25 (43.9)
Muscle ache/pain	18 (33.3)	56 (29.0)	18 (69.2)	30 (52.6)
Dysphagia	12 (22.2)	42 (21.8)	4 (15.4)	7 (12.3)
Insomnia	11 (20.4)	39 (20.2)	6 (23.1)	22 (38.6)
Abdominal pain/bloating	15 (27.8)	31 (16.1)	3 (11.5)	4 (7.0)
Diarrhoea	9 (16.7)	37 (19.2)	1 (3.8)	6 (10.5)
Irritable bowel	5 (9.3)	28 (14.5)	3 (11.5)	4 (7.0)
Constipation	8 (14.8)	21 (10.9)	2 (7.7)	11 (19.3)
Arrhythmia	10 (18.5)	24 (12.4)	3 (11.5)	6 (10.5)
Dyspnea	7 (13.0)	24 (12.4)	3 (11.5)	9 (15.8)
Chronic cough/gastro-esophageal reflux	9 (16.7)	19 (9.8)	2 (7.7)	7 (12.3)
Recurrent chest infections/pneumonia	4 (7.4)	9 (4.7)	3 (11.5)	-
Impaired ability to focus/multitask	7 (13.0)	28 (14.5)	4 (15.4)	13 (22.8)
Cataract/ophthalmological problem	2 (3.7)	15 (7.8)	1 (3.8)	8 (14.0)
